# Identification of African Elephant Polyomavirus in wild elephants and the creation of a vector expressing its viral tumor antigens to transform elephant primary cells

**DOI:** 10.1371/journal.pone.0244334

**Published:** 2021-02-05

**Authors:** Virginia R. Pearson, Jens B. Bosse, Orkide O. Koyuncu, Julian Scherer, Cristhian Toruno, Rosann Robinson, Lisa M. Abegglen, Joshua D. Schiffman, Lynn W. Enquist, Glenn F. Rall

**Affiliations:** 1 Fox Chase Cancer Center, Program in Blood Cell Development and Function, Philadelphia, Pennsylvania, United States of America; 2 RESIST Cluster of Excellence, Institute of Virology at Hannover Medical School, Center for Structural Systems Biology, Hamburg, Germany; 3 Heinrich Pette Institute, Leibniz Institute for Experimental Virology, Hamburg, Germany; 4 Princeton University, Department of Molecular Biology, Princeton, New Jersey, United States of America; 5 Huntsman Cancer Institute, University of Utah, Salt Lake City, Utah, United States of America; Cardiff University, UNITED KINGDOM

## Abstract

Wild elephant populations are declining rapidly due to rampant killing for ivory and body parts, range fragmentation, and human-elephant conflict. Wild and captive elephants are further impacted by viruses, including highly pathogenic elephant endotheliotropic herpesviruses. Moreover, while the rich genetic diversity of the ancient elephant lineage is disappearing, elephants, with their low incidence of cancer, have emerged as a surprising resource in human cancer research for understanding the intrinsic cellular response to DNA damage. However, studies on cellular resistance to transformation and herpesvirus reproduction have been severely limited, in part due to the lack of established elephant cell lines to enable *in vitro* experiments. This report describes creation of a recombinant plasmid, pAelPyV-1-Tag, derived from a wild isolate of African Elephant Polyomavirus (AelPyV-1), that can be used to create immortalized lines of elephant cells. This isolate was extracted from a trunk nodule biopsy isolated from a wild African elephant, *Loxodonta africana*, in Botswana. The AelPyV-1 genome contains open-reading frames encoding the canonical large (LTag) and small (STag) tumor antigens. We cloned the entire early region spanning the LTag and overlapping STag genes from this isolate into a high-copy vector to construct a recombinant plasmid, pAelPyV-1-Tag, which effectively transformed primary elephant endothelial cells. We expect that the potential of this reagent to transform elephant primary cells will, at a minimum, facilitate study of elephant-specific herpesviruses.

## Introduction

More than 175 species and subspecies of the order Proboscidea have inhabited the earth for the last 60 million years [1]. Only three species remain: *Loxodonta africana*, the African savannah elephant; *Loxodonta cyclotis*, the African forest elephant; and *Elephas maximus*, the Asian elephant. All three species are listed in the Appendices of the Convention on International Trade in Endangered Species of Wildlife Fauna and Flora (CITES) as either critically endangered or extremely vulnerable [2, 3]. Habitat fragmentation, increasing human-elephant conflict, and poaching for the illicit trade of elephant ivory, skin, and internal organs have decimated, even eliminated, entire populations of wild elephants in all traditional elephant range countries across Asia and Africa [4–11].

Wild and captive Asian and African elephant populations are further threatened by often-fatal diseases caused by elephant endotheliotropic herpesviruses (EEHVs) [12]. More than 100 cases of mortality attributed to EEHVs have occurred in predominantly young wild and captive Asian elephants in Asia, North America, and Europe [13–26]. Despite their grave consequences, little is known about the molecular biology of these viruses. Pathology upon elephant autopsy is characterized by high-level viremia, cyanosis of the tongue, edema of the head and limbs, lethargy, tachycardia, and hemorrhaging of internal organs, which usually results in rapid death between one and seven days after the appearance of the first clinical signs [17, 27, 28]. In captive African elephants, a dozen known cases of severe disease and/or mortality attributable to EEHVs have been reported. However, the impact of herpesviruses in wild African elephants may be underestimated, because presumption of anthrax infection as the cause of death often precludes field necropsy of unexplained sudden deaths [12, 22, 29–32]. A major obstacle in studying these viruses, including co-infections with elephant gammaherpesviruses (EGHVs) [22, 33, 34], is the paucity of established and well-characterized cell lines that support propagation and enable study of these viruses in elephant cells *in vitro* [12, 30]. No published immortalized elephant cell lines exist [35, 36].

Elephants have a lower incidence of cancer than expected in mammals of their great size and cell number [37]. Immortalized elephant cell lines would be extremely useful tools for exploring the functional consequence of genomic alterations unique to elephants. In addition, they could prove valuable for characterizing mechanisms of cancer resistance in elephants, which might contribute to novel therapeutic approaches for cancer in humans. Genetic loci such as *TP53* (a tumor suppressor gene of which elephants have twenty times more copies than humans), *VRK2* (vaccine related kinase 2), *FANCL* (a RING domain protein mediating E3 ligase activity of the Fanconi anemia core complex, a master regulator of DNA repair), *BCL11A* (B cell lymphoma/leukemia 11A), and *LIF6* (a leukemia inhibitory factor with pro-apoptotic functions) are being explored as important candidate loci for shaping mutation and cancer resistance in elephants, in an effort to understand the effects of genome-wide species differences in the intrinsic cellular responses to DNA damage [38–40].

We had two primary objectives in this study. The first was to assemble a fully annotated and comprehensive collection of tissues from elephant specimens that we expect will be of great value to the veterinary virology community. The second goal was to develop tools to enable establishment of long-lived elephant cell lines that are critically needed for the study of elephant herpesviruses, the development of vaccines and anti-viral therapeutics, and for investigation of the functional consequences of genomic alterations unique to elephants. It is well-known that vectors expressing the simian polyomavirus SV40 large T antigen induce neoplastic transformation and immortalize cells from other species [41]. When characterization of a new elephant polyomavirus, AelPyV-1, (5722 base pairs, first identified in protruding hyperplastic fibrous trunk lesions biopsied from a captive African elephant in Europe), revealed five putative open-reading frames, including the canonical large (LTag) and small (STag) tumor antigen genes [42], we hypothesized that expression of the elephant polyomavirus AelPyV-1 tumor antigens could immortalize elephant primary cells. For perhaps obvious reasons, these cells are infrequently available, difficult to obtain, and challenging to culture. Here, we report the creation of a recombinant plasmid, pAelPy-1-Tag, cloned from a wild isolate of the African Elephant Polyomavirus (AelPyV-1) to establish such cell lines.

## Materials and methods

### Sample collection and permits

Field sample collections were conducted under authorization of Elephants Without Borders Botswana Research Permit/ #WT8/36/4XV(41), Researcher Virginia R. Pearson (GPS-coordinates Chobe National Park, Kasane, Botswana, 17°47’42.83"S, 25°10’16.067"E); Kenya Research Permit #NCST/RRI/12/1/MAS/9, Permittee Virginia R. Pearson, and Kenya Wildlife Service Veterinary Capture and Services Department (GPS-coordinates Samburu National Reserve, Samburu County, Kenya, 0°37’5”N, 37°31’48”E and Keekorok, Masai Mara National Reserve, Narok, Kenya, 1° 35’ 9.00"S, +35° 15’ 6.00"E); South African National Parks Veterinary Wildlife Services (GPS-coordinates Timbavati Reserve, Mpumalanga Province, South Africa, 24°20’07S,31°20’38”E, and Crocodile Bridge, Kruger National Park, South Africa, 25°21’30”S,31°53’32”E); and CENEREST and Agence Nationale des Parcs Nationaux Gabon, (GPS-coordinates Minkebe NP 1°40’47”N,12°45’34”E; Mwagna NP 0°36’N,12°42’E; Ivindo NP 0.088°N, 12.63°E; Pongara NP 0°07’N,9°38’E; Loango NP 2°10’S,9°34’E; Moukalaba Doudou NP 2°26’S, 10°25’E). Samples were imported into America under authorization of United States Veterinary Permit for Importation and Transportation of Controlled Materials and Organisms and Vectors #11798, Permittee Virginia R. Pearson; San Diego Zoo Global CITES/USFWS Import Permit # 13-US727416/9 and #10-US727416/9; International Elephant Foundation CITES/USFWS Import Permit #17US09806C/9; Botswana CITES Export Permit #0202118; Gabon CITES Export Permit #0785; Kenya CITES Export Permit # 008624/OR82872; and South Africa CITES Export Permit #1087577. Protocols for elephant immobilization and sample collection were reviewed by San Diego Zoo Global Institutional Animal Care and Use Committee IACUC #11–002. Samples from within the United States were collected according to participating zoos’ Animal Welfare and Research Committees and Association of Zoos and Aquariums Elephant Research and Necropsy Protocol. Wild African elephants were immobilized by qualified, experienced field veterinarians using etorphine hydrochloride (M99). 5 mm punch biopsies were excised from trunk nodules and immersed in 2.5 ml RNALater Reagent (Qiagen). Incisions were treated with iodine and oxytetracycline. Diprenorphrine hydrochloride or naltrexone reversed sedation within 90 sec. Saliva samples from immobilized, or semi-captive wild or captive elephants in Africa and America were collected using 16 in polyester swabs (Puritan Medical Corporation) until saturated then immersed in 2 ml RNAprotect Cell Reagent or RNALater (Qiagen).

### DNA Extraction

DNA was extracted from trunk nodule biopsies, saliva, tissues, and cultured cells using Qiagen DNEasy Blood and Tissue Kit according to the manufacturer’s instructions with the modification of extended overnight incubation at 56°C, followed by 10 min incubation at 95°C. Archived (FFPE) biopsies, were re-embedded in fresh paraffin after which 8 mm microtome slices per biopsy were incubated in xylene at room temperature for 5 min shaking and rinsed with 100% EtOH a total of five times, prior to DNA extraction.

### Polymerase chain reaction for AelPyV-1 genes

For each 25 μl PCR reaction, we used 2 μl elephant DNA, 12.5 μl GoTaq G2 Hot Start Green Master Mix (Promega), 8 μl sterile nuclease-free H_2_O, and 1.25 μl each forward and reverse primers specific for five AelPyV-1 genes. Reactions were run on an Eppendorf MasterCycler thermocycler program: 95°C for 2 min; 45 cycles of 95°C for 40 sec, 50°C for 45 sec; 73°C for 60 sec; final extension 78°C for 7 min. 25 μl of each PCR reaction was run on 2% agarose electrophoresis gels with ethidium bromide or GelRed nucleic acid stain (Phenix Research Products). Bands of expected size were purified using QIAquick Gel Extraction Kit (Qiagen).

### Sequencing

Primer extension sequencing was performed by Genewiz, Inc (South Plainfield, NJ) using Applied Biosystems BigDye version 3.1. The reactions were then run on Applied Biosystem’s 3730xl DNA Analyzer. Sequences were entered into NCBI BLAST program and compared to known AelPyV-1 gene sequences in GenBank accession #NC-0225191.9.

### Cloning PAelPyV-1-Tag from wild African elephant trunk nodule biopsy

A 50 μl PCR reaction was prepared using 2 μl DNA template from BW1MaleNOD1 (concentration 174 ng/μl), 5 μl NEB Buffer #2, 1 μl each forward and reverse primer targeting AelPyV-1-Tag, 1 μl DNTP, 0.5 μl High Fidelity Taq (Roche), and 39.5 μl water. This sample was then run on an Eppendorf Mastercycler Model 5544 program: preheated 95°C for 1 min, twenty cycles of 95°C for 30 sec, 70°C for 30 sec, 72°C for 30 sec, and 95°C for 30 sec. 5 μl of this reaction was run on a 1% agarose gel with ethidium bromide to verify expected band size of 2265 bp. This was followed by purification of the entire PCR product using QIAquick PCR Purification Kit, and elution of insert DNA in 30 μl Buffer EB.

The entire 30 μl of insert DNA was digested in a 100 μl reaction, containing 5 μl restriction enzyme HindIII (20U/μl), 5 μl restriction enzyme NOT1 (20U/μl), 10 μl NEB #4, and 50 μl ddH_2_0. The insert digest was incubated at 37°C shaking 2 h, stored @ 4°C, then purified using QIAquick PCR Purification Kit. Insert DNA was eluted in 30 μl Buffer EB. In a separate control 100 μl reaction, 11 μl of the plasmid backbone pEGFP-N1 @ 2 μg/μl,(accession #U55762) was digested with 5 μl HindIII (20 U/μl), 5 μl NOT1 (20 U/μl), 10 μl NEB CutSmart #4, and 69 μl ddH_2_0. The control digest was incubated at 37°C shaking for 2 h then stored at 4°C. The entire backbone control digest was loaded onto a 1% agarose gel stained with SyberGreen (1 μl of 1:100,000 stock). The expected ~4 kb band was cut out and purified using QIAPrep Spin MiniPrep Kit. Backbone DNA was eluted in 30 μl Buffer EB. Digested insert DNA and backbone DNA were quantified and normalized to 100 ng/μl for 20 μl ligation reactions. Ligation reaction #1 (insert + backbone) contained 11.0 μl digested and purified insert DNA (174.2ng/μl), 1.43 μl digested and purified backbone DNA, 2 μl NEBT4 ligation buffer, 1μl NEB T4 ligase and 14.57 μl H_2_O. Ligation reaction #2 (control-backbone only) contained 1.43 μl digested and purified backbone DNA, 2 μl NEBT4 ligation buffer, 1μl NEB T4 ligase, 15.57 μl H_2_O. Each mixture was incubated shaking overnight at 16°C. 10 μl of each ligation reaction was transformed into 50 μl NEB DH5A competent bacteria and incubated on ice for 30 min, heat shocked for 30 sec in a 42°C water bath and then incubated on ice for 1 min. Then 500 μl SOC recovery media (Sigma) was added to each tube and the mixtures were incubated for 1 h at 37°C with vigorous shaking. Bacteria were pelleted (3000 rpm for 4 min), the supernatant was removed and each pellet was resuspended in 50 μl SOC media. The entire volume of each was streaked onto pre-warmed Kanamycin-resistant LB plates using glass beads and incubated overnight at 37°C shaking. Ten colonies from insert+backbone LB plate only were picked for PCR. Each 25 μl PCR reaction contained 1.25 μl AelPyV-1-LTag+ HindIII forward primer, 1.25 μl AelPyV-1-LTag+NOT1 reverse primer, 12.5 μl Promega Master Mix and 10 μl ddH_2_O. This was then run on Eppendorf Mastercycler Model 5544 program: 95°C for 2 min, 45 cycles of 95°C for 40 sec, 50°C for 45 sec, 73°C for 60 sec, then 78°C for 7 min. PCR products were run on 1.5% agarose gel with EtBr, and analyzed for expected band size of 2265 bp. 10 PCR-positive colonies were again picked and added to each of 10 ml falcon tubes containing 5 ml LB and 5 μl Kanamycin and incubated O/N at 37°C shaking. 1 ml of each clone was aliquoted and stored at 4°C for later use and plasmid DNA was isolated from each remaining volume using QIAprep Spin Miniprep Kit (Qiagen). 500 ng (2 μl) of plasmid DNA from each clone and 500 ng (2 μl) parental plasmid pEGFP-N1 were digested in 20 μl reactions with 0.5 μl HindIII (20 U/μl), 0.5 μl Not1 (20 U/μl), 2 μl NEB Buffer 4 and 15 μl H_2_O. The digests were incubated at 37°C for 2 h shaking. Entire digests were loaded onto 1% agarose gel with EtBr and analyzed for expected band size of 2265 bp. Appropriate size gel bands from Clones #2, #4, #7 and #8 were selected, cut out and plasmid DNA was purified using Qiagen QIAquick Gel Extraction Kit and eluted in 50 μl Buffer EB. Plasmid DNA from these clones were sequenced with standard primers (CMV forward and SV40 reverse; Genewiz) to verify the presence and integrity of the AeIPyV-1 early region sequence. Positively sequenced colonies from Clones #4 and #7 were grown in 100 ml LB plus 10 μl Kanamycin and incubated O/N at 37°C shaking. The bacterial pellets were harvested by centrifugation and plasmid DNA was isolated using Qiagen Plasmid Maxi Kit. Plasmid DNA was eluted in Buffer EB and stored at -80°C for transfection experiments. 1 ml of all clone LB stocks were frozen at -80°C in a 1:1 mixture of LB and glycerol.

### Cloning mCherry-tagged Tag

AelPyV1 early region DNA was amplified from elephant pAelPyV-1-Tag Clone #4 using Platinum Pfx DNA polymerase (ThermoFisher) in a 25 μl reaction (2X Amplification Buffer, 1x Enhancer, 0.3 mM dNTP (final), 1 mM MgSO_4_ (final), 1 U platinum pfx, 0.4 μM each forward and reverse primer, and 10 ng plasmid DNA. This was then run on ThermoFisher SimpliAmp thermocycler program: 98°C for 5 min, followed by 35 cycles of 96°C for 30 min, 62°C for 30 min, and 72°C for 3 min, followed by a final step at 72°C for 10 min. 25 μl of PCR product was run on a 2% agarose gel with ethidium bromide. Expected bands of size of ~2.3 kb were gel purified using QIAquick Gel Extraction reagents with SpinSmart PCR Purification and Gel Extraction Columns (Denville Scientific). The purified PCR product and 0.5 μg backbone (pcDNA3.1+ with Kozak consensus sequence (GCCACC) and mCherry fluorescent tag (Genscript custom clone) inserted in the multiple cloning site using EcoRI and XhoI) were digested in 50 μl volumes using 4 U XhoI (NEB) and 4 U XbaI (NEB) and 1X CutSmart Buffer (NEB) each. Digests were incubated at 37°C for 2 hrs. The entire volumes were loaded on a 2% agarose gel and the appropriate bands (LTA ~2.3 kb, pcDNA3.1+ ~6.1 kb) cut out and purified using QIAquick Gel Extraction reagents with SpinSmart PCR purification and gel extraction columns. Digested PCR product and backbone DNA were quantified and normalized to 10 ng/μl for 20 μl ligation reactions. Each ligation contained 10 ng of digested backbone and 0, 10 or 100 ng of digested PCR product with 1X T4 ligase buffer and 5 U T4 Ligase (ThermoFisher). These ligations were incubated at room temperature overnight; 5 or 15 μl of the overnight ligation was transformed into 50 μl DH5alpha competent bacteria and incubated on ice for 45 min, heat shocked 90 sec in a 42°C water bath, then incubated on ice for 5 min; then 700 μl SOC media (Sigma) was added to each tube and the mixture was incubated in 37°C shaking incubator for 1 h. Bacteria were pelleted (3000 rpm for 4 min), the supernatant was removed, and the pellet was resuspended in 200 μl of SOC media. The entire volume was spread on LB+Amp plates using glass beads and incubated overnight at 37°C. Single colonies were selected and inoculated into 5 mL LB with carbenicillin and incubated overnight at 37°C with shaking. Ten μl of each overnight culture was spotted on LB+Amp plates and plasmid DNA was isolated from the remaining volume using PureLink Quick Plasmid Miniprep Kit (ThermoFisher). Plasmids were sequenced to verify the presence and integrity of the AeIPyV-1 early region sequence. A single clone was selected and used in a maxi-prep (Zymo Research) to isolate plasmid DNA). mCherry expression in African elephant fibroblasts was documented using the Evos FL (ThermoFisher Scientific).

### Transfection

100 μl transfection reaction contained 82 μl Amaxa Nucleofector Solution plus 18 μl supplement (Amaxa^R^ HUVEC Nucleofector^R^ Kit for Human Umbilical Vein Endothelial Cells, Lonza), 4-5x10^5^ pelleted elephant endothelial or epithelial cells were resuspended in 50 μl EGM^R^2 Bullet Kit media (LONZA), 6 μg DNA (actual 2 μl at 3050ng/μl of Clone #7) and pMaxEGFP @ 0.5μg/μl per cuvette. We used Amaxa^R^ Nucleofector^R^ Program A-034 (Lonza) for endothelial cells and S-005 (Lonza) for epithelial cells and plated the transfected cells immediately onto untreated cell culture six-well plates (Nunc) with 3 ml prewarmed EGM^R^2BulletKit^R^(Lonza) plus 36 μl Genetecin Selective Antibiotic G418 at 50 mg/ml (Life Technologies).

Transfection of African elephant fibroblasts (San Diego Zoo Global) with pcDNA3.1+mCherry constructs was performed using the Neon Transfection System (ThermoFisher Scientific). 1x10^6^ fibroblasts were transfected with either 2.5 μg pcDNA3.1+mCherry or 2.5 μg pcDNA3.1+mCh-LTag in 100 μl of Neon Resuspension Buffer R. Neon program used for transfection was 2 pulses at 1300 V/pulse with 20 ms pulse width. Immediately after transfection, cells were transferred to T25 flasks containing 5 ml of DMEM + 10% FBS. 18 h post transfection, live cells were examined and imaged for mCherry and mCherry-Tag expression using a fluorescent microscope (Evos FL, ThermoFisher Scientific).

### Western blot

pcDNA3.1+mCherry and mCherry-LTag transfected African elephant fibroblasts (San Diego Zoo Global) were harvested and pelleted 24 h post-transfection. The cells were transfected using the Neon transfection protocol described above. Pelleted cells were frozen at -20°C overnight. Pelleted cells were then thawed and lysed using Cell Signaling Lysis buffer supplemented with Protease/Phosphatase Inhibitor Cocktail (Cell Signaling). 60 μg of total protein lysate from each sample was loaded and run on a 4–12% Bis-Tris Bolt gel (ThermoFisher Scientific). Proteins were transferred to a PVDF membrane using the iBlot2 (ThermoFisher Scientific). Membrane was blocked and antibody diluted in Starting Block (ThermoFisher Scientific). Mouse monoclonal mCherry antibody, HRP conjugated (Origene, clone OTI10G6) was used for immunodetection. Substrate used for detection was Western Lightning Ultra (PerkinElmer). Signal was detected with a Bio-Rad ChemiDoc Gel Imager.

### Reverse transcriptase PCR

RNA was extracted using Qiagen RNeasy Mini Kit according to the manufacturer’s protocol. We treated the RNA to remove all contaminating genomic DNA using Turbo DNA-free TM Kit (Invitrogen) according to manufacturer’s directions using a minimum of 1,000 ng RNA resuspended in 50 μl RNase-free water, 5 μl Turbo Buffer @10x, and 1 μl TurboDNase. We then used SuperScript IV One-Step RT-PCR System (Invitrogen) to perform reverse transcriptase (RT) polymerase chain reaction (RT-PCR). We included a no RT control for each sample. For each 50 μl RT-PCR reaction, we used 25 μl 2X Platinum Super-Fi RT-PCR Master Mix, 2.5 μl each forward and reverse primers at 10 μM concentration, 0.5 μl SuperScript IV RT Mix, template RNA at 1000 ng per reaction and nuclease-free water to 50 μl. The sample was amplified using Eppendorf MasterCycler program as follows: reverse transcription at 57°C for 10 min, followed by RT inactivation/initial denaturation at 98°C for 2 min, followed by 40 cycles of 98°C for 10 sec, 65°C for 10 sec, 72°C for 30 sec, followed by a final extension of 72°C for 1 min. RT-PCR products were the run on 1.5% electrophoresis gel. Bands of expected size were purified using Qiagen QIAquick Gel Extraction Kit and sequenced.

### Cell culture

Elephant umbilical cords and amniotic sacs from live captive births were shipped within 24–48 h post birth in accordance with Association of Zoos and Aquariums Elephant Research and Necropsy Protocol. Procedures for Primary Elephant Cell Culture and Elephant Herpesviruses Cell Culture in the Enquist Lab was approved by Princeton University Institutional Animal Care and Use Committee Protocol #1819. The study involves by-product material of animal origin (elephants), and does not therefore fall under federal regulations governing research involving live vertebrate animals at Fox Chase Cancer Center. Elephant endothelial and epithelial primary cells were extracted from umbilical cords and amniotic sacs, respectively. Briefly, umbilical cords were infused with 0.2% collagenase (Sigma), incubated at 37°C for 30 min, flushed with warm PBS into endothelial cell growth medium EGM^2^BulletKit^™^ (Lonza) containing penicillin/streptomycin and antimycotic Normocin^™^ at 50 mg/ml (Invivogen) and plated into T25 flasks or 6-well plates. The amniotic sac was cut into 3 cm squares and incubated in 0.25% trypsin at 37°C for 90 min, collecting cells and adding fresh trypsin at 30 min intervals. Epithelial cells were plated in HamsF12 (Lonza)+pen/strep+Normocin^™^+10% FBS. African elephant fibroblasts used in transfection experiments with mCherry vectors were provided by San Diego Zoo Global. Fibroblasts were cultured in DMEM + 10% FBS.

### Immunofluorescence

Cells were washed with D-PBS, and fixed with 4% PFA/D-PBS for 10 min at 37°C. Thereafter, the cells were washed with D-PBS, and permeabilized with 0.1% Triton X100/D-PBS for 20 min at RT, and then washed again three times with D-PBS; blocked with 3% BSA/D-PBS for 1 hr at RT, and incubated with anti-von Willebrand Factor antibody produced in rabbit (1:200; Sigma) in 3% BSA/D-PBS for 1 h at RT. After washing, the cells were then incubated with AlexaFluor 488 (1:200 plus 1:500 Hoescht nuclear stain (Sigma)), washed a final time, and mounted in CITIFLUOR (Electron Microscopy Sciences) and imaged on Olympus BX50.

## Results

### Identification of AelPyV-1 in Wild African Elephants

**Fig 1:**
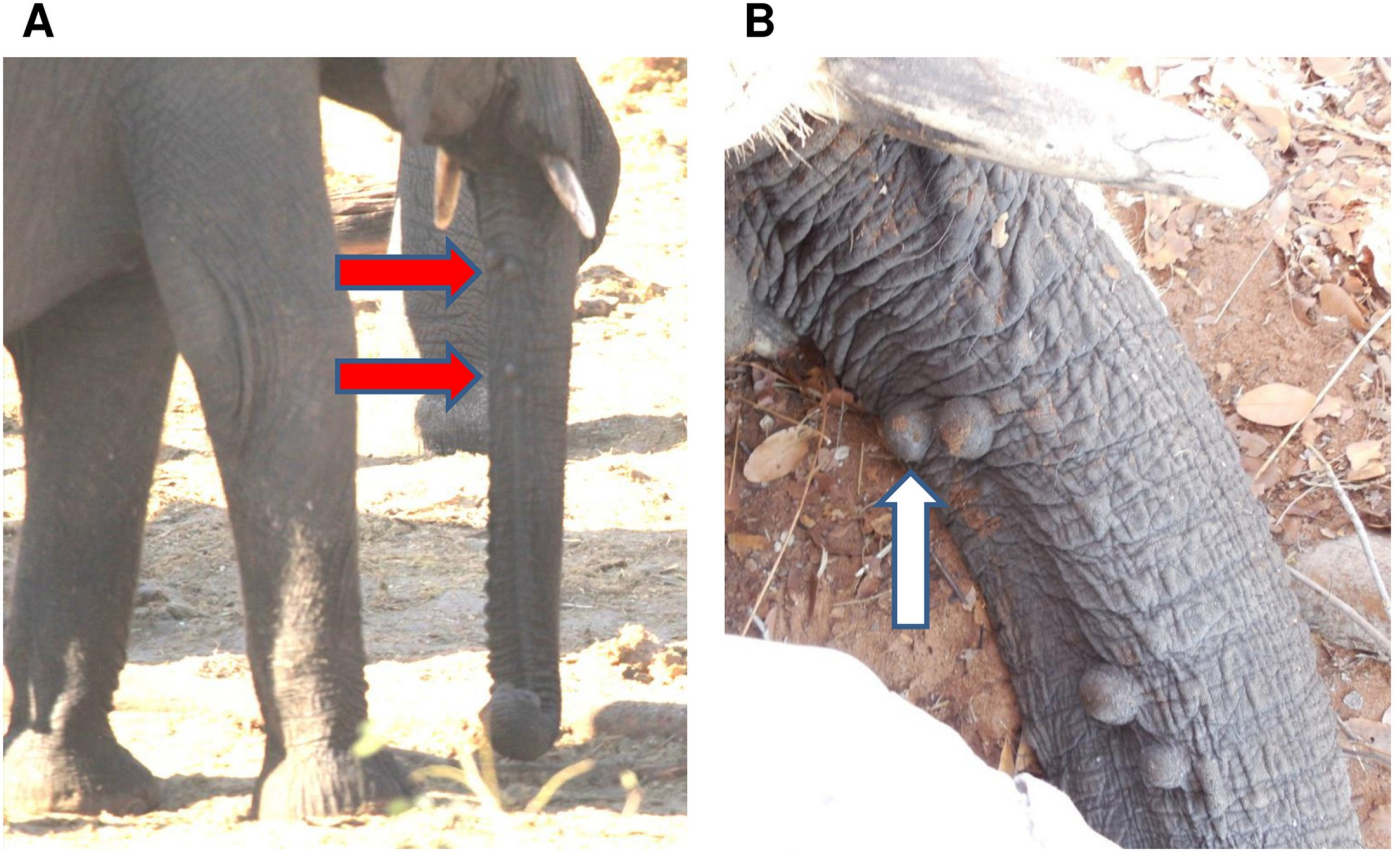
Nodule location. Trunk location (A) and gross appearance (B) of nodules present on a wild juvenile African elephant in Botswana (BW1 Male NOD 1–4). Samples collected under Elephants Without Borders Botswana Research Permit/ \#WT8/36/4XV(41). Location: Kalwesi Water Hole, Chobe National Park, Botswana. Red arrows indicate nodular position on the trunk; white arrow indicates the nodule from which the DNA was extracted to create the recombinant plasmid.

**Fig 2:**
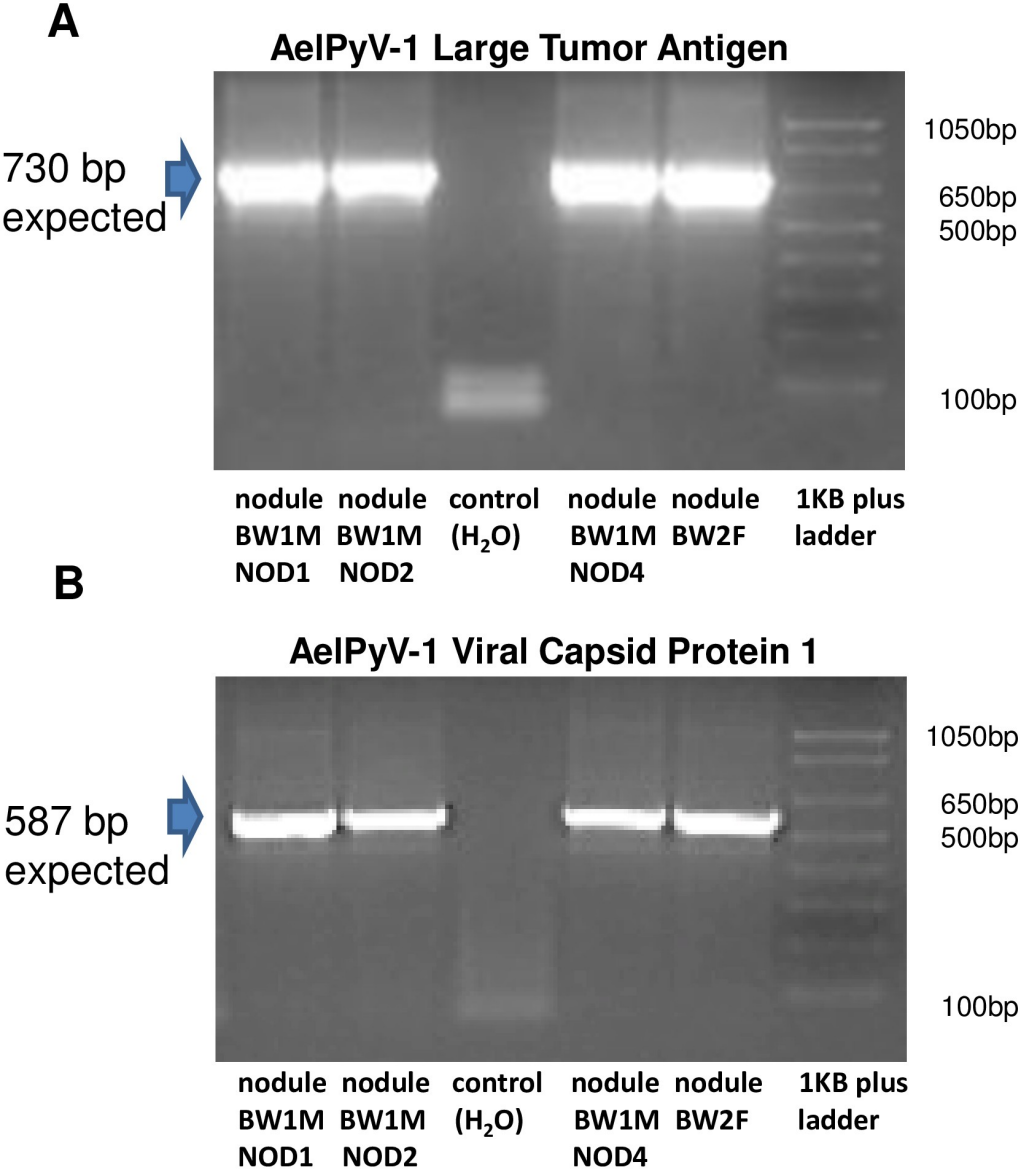
PCR analysis of biological specimens. PCR analysis of elephant trunk nodules in Botswana and South Africa to detect: A) AelPyV-1 large tumor antigen (expected size 730 bp) and B) viral capsid protein 1 (expected size: 587 bp).

Using primers specific for five AelPyV-1 genes (Table 1), we screened an extensive archive of wild and captive African and Asian elephant DNA. The first set of samples that were screened consisted of DNA extracted from biopsies of trunk nodules collected from seven wild African elephants in Botswana (2013) and Kenya (2011) that had previously tested positive for one or more species of EEHVs and EGHVs as part of an independent study [43–45] (Fig 1). Using standard PCR methods and Sanger sequencing, we identified nucleotide sequences in nodule biopsies from six of seven of these wild elephants that matched genes of the index AelPyV-1 genome (Genbank Accession number KF147833.1), including those encoding the large tumor antigen (AelPyV-1-LTag), viral capsid proteins (AelPyV-1-VP1 and AelPyV-1-VP2), and regulatory non-coding proteins (AelPyV-1-NC1 and AelPyV-1-NC2; Fig 2, Table 2). The DNA extracted from the nodule biopsy of one of these six elephants, a juvenile male elephant in Botswana (sample isolate BW1maleNOD1; Fig 1), was subsequently used to create a recombinant plasmid, pAelPyV-1-Tag. The nucleotide sequence segment of the AelPyV-1-Tag gene identified in this wild elephant from Botswana has been deposited into Genbank (accession: MH934191, 709 bp DNA linear VRP 20-FEB-2019 African elephant polyomavirus 1 isolate BW1maleNOD1 large tumor antigen gene, partial cds).

**Table 1:** Primers used in this study.

AelPyV-1 Large Tumor Antigen (PCR product size: 730 bp) forward–ACACAGCCATCCATGTGGTCT reverse---CAGGAGGGTTCGAAGCAAGT
AelPyV-1 Viral Capsid Protein 1 (PCR product size: 587 bp) forward-AGTGGAACCAGAAGGCATCG reverse--CCTGTCCCTTGTCACCTGTC.
AelPyV-1 Viral Capsid Protein 2 (PCR product size: 790 bp) forward-GCATCTCAGCTAGAGGCGTT reverse–GGACGTTTTCCGGTTTTCGG
AelPyV-1 Non-coding Regulatory Protein 1 (PCR product size: 345 bp) forward-ATTACCGCAGGTTTGGCTGT reverse--CCAGGCACTGGTTAATGTGC
AelPyV-1 Non-Coding Regulatory Protein 2 (PCR product size: 814 bp) forward–TCTGCTTGGCACTGTTACCC reverse--AGGGGAGGGGGTTTATTGGA
AelPyV-1-LTag ends (HINDIII and NOT1 restriction sites; PCR product 2225 bp) forward—GCAAGCTTATGGACAGGGCTTTGAATAGAG reverse—GGATTCAAGAGACTTCACAGACTAAGCGGCCGCGC
AelPyV-1-LTag cDNA forward-ACCATCATGAAGGAGTTGAATAGGT reverse- GCTGCAGAGGAAGAACCGT
AelPyV-1-LTag- mCherry fluorescent tag. forward-TATCCGCTCGAGATGGACAGGGCTTTGAATAGAGAAGACAG, reverse--TCCTAGTCTAGATTAGTCTGTGAAGTCTCTTGAATCCGTTTCCG

**Table 2:** Trunk nodule biopsies from wild elephants (collected by Virginia R. Pearson).

COHORT 1	AelPyV-1-LTag	AelPyV-1-VP1	AelPyV-1-VP2	AelPyV-1-NC1	AelPyV-1-NC2
**Botswana (2013)**					
BW1 Male NOD	+	+	+	+	+
BW2 Fem NOD	+	+	+	+	+
**Kenya (2011)**					
CalfGM Fem NOD	-	-	-	-	-
CalfMT Fem NOD	+	+	+	+	+
HIM Fem NOD	+	+	+	-	+
CalfMM Male NOD	+	+	+	-	+
MM Fem NOD	+	+	+	-	+

### Creation of a recombinant plasmid encoding AelPyV-1 T antigens

**Fig 3:**
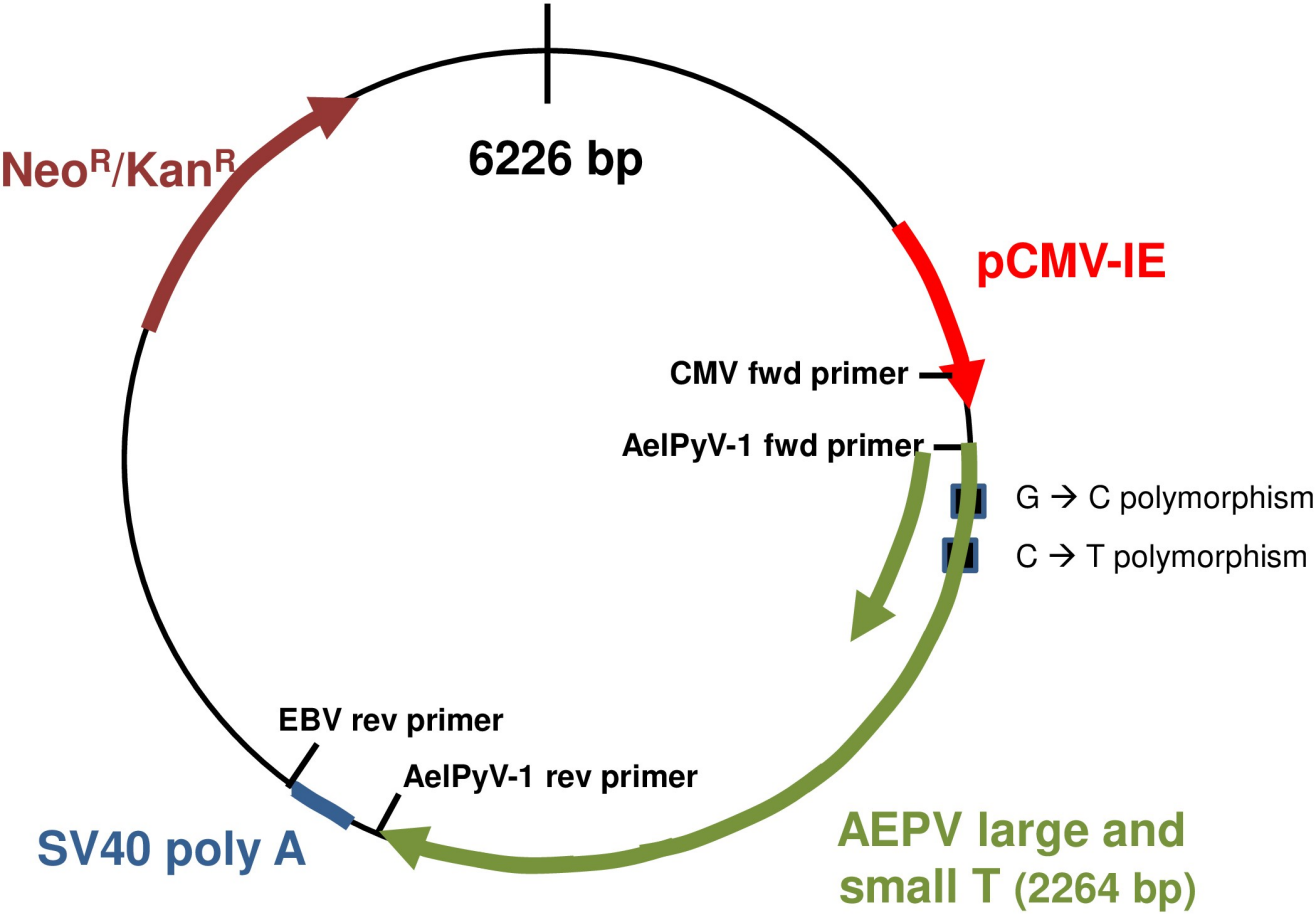
Cloning strategy. The T antigen-encoding region was amplified from elephant BW1 MaleNOD1, then inserted into the plasmid pEGFP-N1 (accession #U55762). DH5A competent bacteria were transformed and grown on kanamycin-resistant LB and amplified in a PCR reaction. PCR products were analyzed for expected band size of 2265 bp. Appropriate size gel bands were sequenced with standard primers (CMV forward and SV40 reverse to verify the presence and integrity of the AeIPyV-1 early region sequence.

We used standard molecular biology techniques to clone the AelPyV-1 early region (2264 base pairs), spanning the large and small tumor antigen regions, from DNA extracted from BW1maleNOD1. (A full genomic map of the AelPyV-1 virus can be found in 42, where it was originally reported). This amplified region was then inserted into the high-copy plasmid vector, pEGFP-N1, with an antibiotic selection marker. The GFP cassette of the backbone vector was intentionally deleted, because tags can interfere with normal protein function. This recombinant plasmid was named pAelPyV-1-Tag (6226 base pairs) (Fig 3).

**Fig 4:**
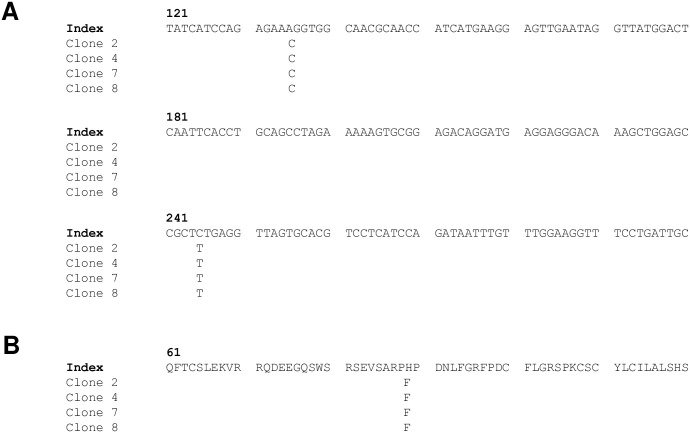
Predicted amino acid alignment. PAelPyV-1-Tag clones 2, 4, 7 and 8 partial nucleotide (A) and amino acid (B) alignment compared to the AelPyV-1 index genome (GenBank accession # NC-0225191.9).

Using universal primers (forward primer: CMV; reverse primer: SV40 polyA) flanking the inserted gene, we sequence validated 4 of 8 pAelPyV-1-Tag clones against the AelPyV-1 early region positions 3454 to 5722 of the published index genome of African Elephant Polyomavirus (GenBank accession # NC-0225191.9) A 555 base pair segment between positions 4281 and 4836 was not sequenced. All sequenced clones of pAelPyV-1-Tag contained the same two polymorphisms that differed from the index genome at positions 5591 and 5478 (Fig 4).

### AelPyV-1 tissue affinity survey

To test whether AelPyV-1 was restricted primarily to nodules, we screened a second set of DNA samples consisting of archived formalin-fixed paraffin-embedded (FFPE) trunk nodule biopsies collected from nine wild African elephants imported from Zimbabwe to the US in 1982–83 (courtesy Elliott Jacobson, DVM, University of Florida School of Veterinary Medicine, USA) [46]. PCR and Sanger sequencing confirmed nucleotide sequences that matched segments of the AelPyV-1-LTag gene in each of the nine FFPE samples (Table 3). We did not test for presence of EEHVs and EGHVs in these archived FFPE samples.

**Table 3:** FFPE Trunk nodule biopsies (preserved by Elliot Jacobsen, DVM).

COHORT 2	AelPyV-1-LTag
Zimbabwe (1982–83)	
Nautilus 80–31 NOD	+
Nautilus 83–345 NOD	+
Nautilus 84–86 NOD	+
Nautilus 86–196 NOD	+
Nautilus 84–290 NOD	+
Nautilus 86–441 NOD	+
Nautilus 89–290 NOD	+
Nautilus 121 NOD	+
Nautilus 133 NOD	+
Nautilus 133A LUNG	-

Additionally, to further investigate the tissue affinity of the elephant polyomavirus, we screened a third, larger set of samples consisting of DNA extracted from the saliva of 57 wild and 26 captive African elephants (including saliva samples matched to trunk nodule biopsies from three of the wild African elephants tested for AelPyV-1 genes); from the saliva of 41 captive Asian elephants; and from blood, serum, non-nodular skin warts, and internal organ biopsies from 2 wild and 5 captive African elephants, and from 6 captive Asian elephants (Table 4). Nodules were visually distinguished from warts based on gross morphology: nodules were round, circumscribed, predominately on the trunk, and often of a different appearance than the surrounding skin. Interestingly, when PCR and Sanger sequencing was performed using primers specific for only AelPyV-1-Tag, PCR products were detected only in saliva samples from just 8 of the 124 elephants surveyed (Table 5). Of the eight positive saliva samples, three came from African elephants, and were also associated with trunk nodules. Importantly, nearly all saliva samples from 124 African and Asian elephants tested in this third set had previously tested positive for at least one species or subspecies of EEHVs or EGHVs as part of the same independent study [43–45]. All other non-nodule tissues tested were negative for AelPyV-1. We conclude that this elephant polyomavirus is generally restricted to skin-associated nodules.

**Table 4:** Complete set of Cohort 3 specimens (collected by Virginia R. Pearson).

	# Saliva Specimens	Other Tissues
BOTSWANA L. africana	22	0
GABON L. cyclotis	25	0
KENYA L. africana	7	1: Lung
SOUTH AFRICA L. africana	3	1: Ear warts
USA L. africana	26	5: Ear warts, organs, blood, serum
USA E. maximus	41	6: Organs, blood, serum
ZIMBABWE L. africana	0	1: Lung FFPE

**Table 5:** Aggregated percentage of samples positive for AelPyV-1.

Samples Collected	# Positive for AelPyV-1	Percent
Trunk nodule biopsies	15 of 18 Elephants	83%
Saliva	8 of 124 Elephants	6%
Blood, serum, skin warts, internal organ biopsies	0 of 12 Elephants	0%

One caveat of this extensive survey to screen for AelPyV-1 in African and Asian elephants is that, for practical limitations of biopsy collections of animals in the wild, we were unable to collect adjacent normal skin samples from elephants with virus-positive trunk nodules. We were also not able to collect internal organ biopsies from deceased elephants exhibiting characteristic trunk nodules premortem.

### Establishment of transformed elephant cell lines

**Fig 5:**
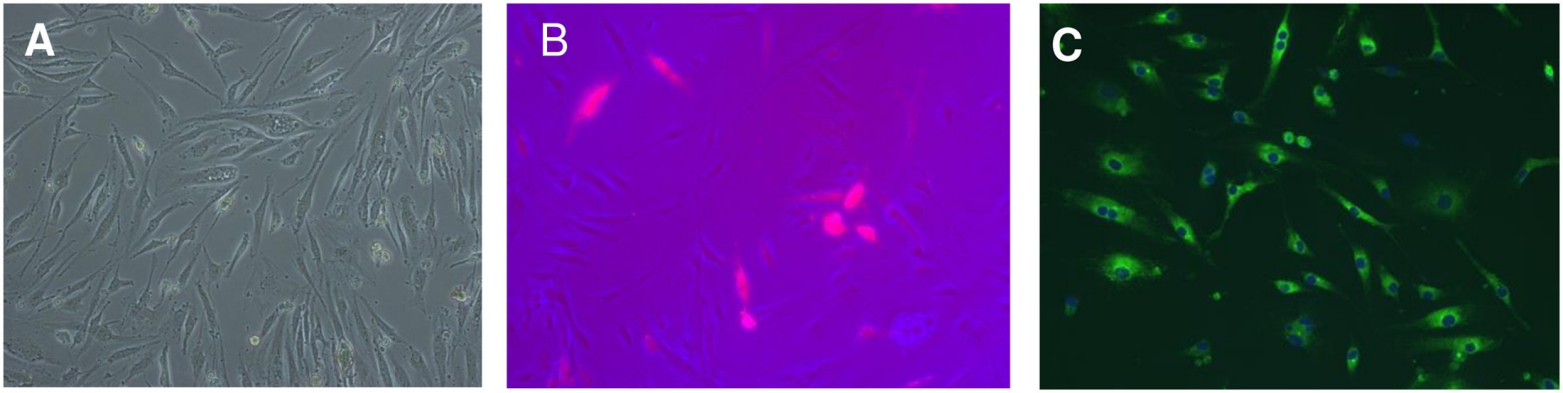
Successful transfection of elephant endothelial cells. **A**: Duncan P.1 umbilical cord primary endothelial cells. **B:** #1DuncanAMAXA P.4 endothelial cells expressing pMaxGFP. **C:** #1DuncanAMAXA cells at 105 days, immunostained for vonWillebrand Factor. Photos taken with an Olympus BX50, 20x original magnification.

We next transfected elephant primary endothelial cells with this recombinant plasmid pAelPyV-1Tag, along with a separate plasmid, pMaxGFP (Lonza Nucleofector Technology), to ascertain efficiency. The primary endothelial cells were derived from the umbilical cord of an Asian elephant named Duncan, born in 2014 at Houston Zoo, Houston, TX USA (Fig 5A). Prior to transfection, we confirmed that Duncan primary endothelial cells were negative for AelPyV-1 (using primers for the AelPyV-LTag, STag, VP1, P2, NC1, and NC2 genes) as well as for elephant endotheliotropic herpesviruses (using primers for EEHV Ter and Pol genes).

As a control for transfection efficiency and potential false-positive results possibly attributable to GFP interference, a separate transfection of Duncan primary endothelial cells was performed using only florescent marker plasmid pMaxGFP. Untransfected Duncan cells of the same passage number were plated for counting the number of passages to cellular senescence. At 24 hours post transfection, GFP expression was observed by fluorescent microscopy in pAelPyV-1-Tag plus pMaxGFP (designated #1DuncanAMAXA) and pMaxGFP-only transfected cells, indicating greater than 50% transfection efficiency.

Transfected cells were cultured in endothelial cell growth media for one week before adding antibiotic (G418) to eliminate untransfected cells. pMaxGFP-only transfected cells died several days after addition of antibiotic G418 while the cells transfected with pAelPyV-1Tag plus pMaxGFP (#1DuncanAMAXA) continued to proliferate under continuous G418 antibiotic selection. We noticed a slower than normal cell doubling time of these cells compared to non-transfected controls. Therefore, at 14 days post-transfection, additional early passage Duncan primary endothelial cells were added to the #1DuncanAMAXA transfected cells at a 4:1 ratio, as it is known that some cells require a critical density to proliferate well. In addition, the FBS concentration was increased to 10% to encourage cell growth. The non-transfected feeder cells were subsequently eliminated by G418 selection.

GFP expression in the transfected cells was observed by fluorescent microscopy throughout ~150 days and seven passages under continuous G418 antibiotic selection pressure (Fig 5B). By comparison, untransfected Duncan primary endothelial cells grown in parallel reached senescence and died after ~17 passages over just 70 days. At 105 days post transfection, the transfected cells were confirmed as endothelial in origin by using an antibody to von Willebrand Factor (Fig 5C). At 150 days post transfection, we confirmed the presence of the AelPyV-1 Tag transgene in cellular DNA homogenates by PCR and Sanger sequencing verification. The then presumed transformed cells were frozen in liquid nitrogen. Subsequent recovery and regrowth did not impact GFP expression. As a further test against potential false-positive results, we performed new transfections of Duncan primary endothelial cells; in these replicate experiments, we observed that pAelPyV-1Tag transfected cells continued to proliferate under G418 selection, while those tranfected with an empty vector died after G418 selection.

### Expression of large and small T antigen RNA in transfected cells

**Fig 6:**
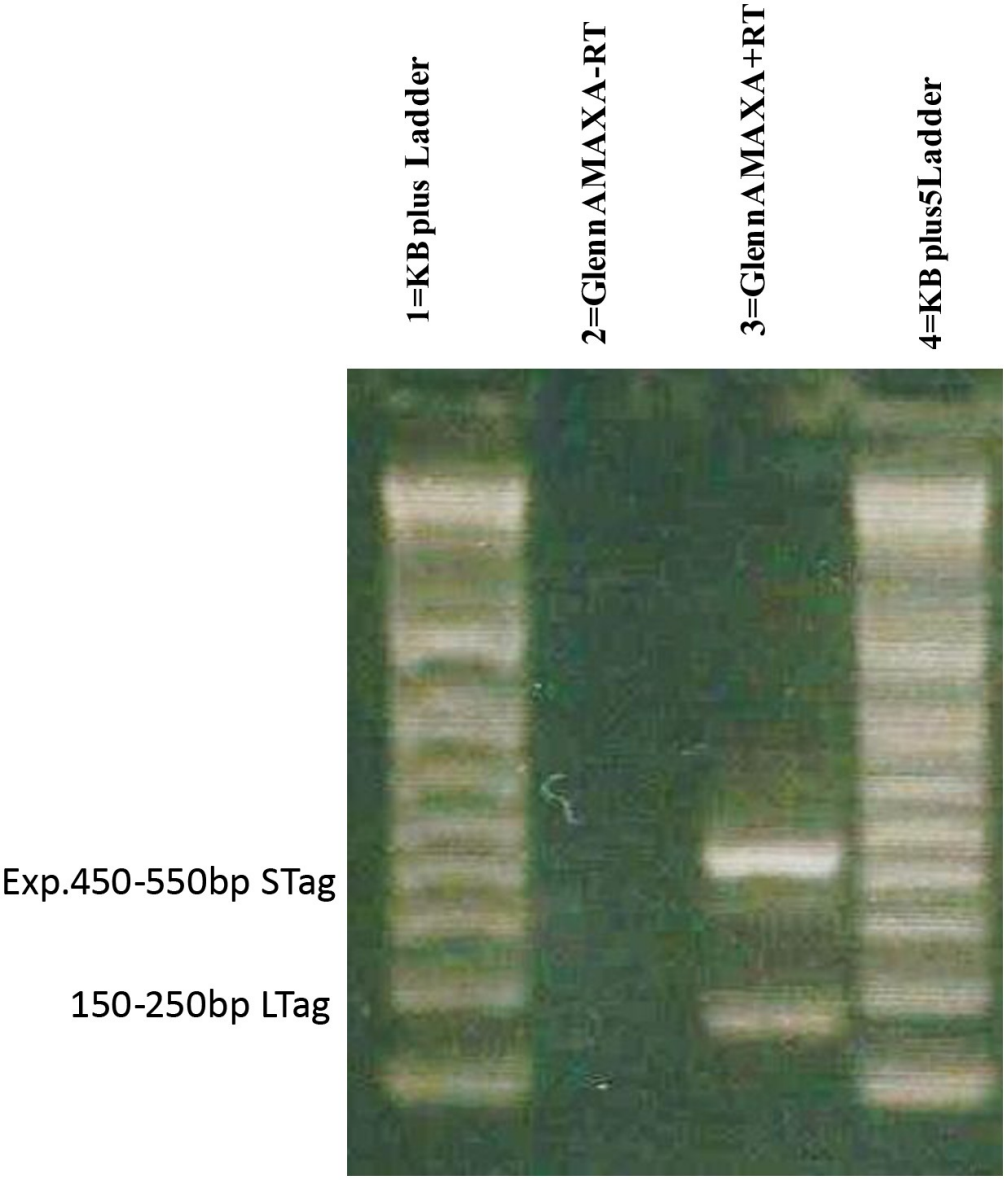
Detection of PCR products from other elephant primary endothelial cultures. Amplification of both AelPyV-1 LTag and STag DNA sequences from endothelial cells derived from an African elephant (Fitz, born 2019, Louisville, KY USA).

We next wished to verify transcription of the large T and small T antigens in the transformed cells. pAelPyV-1-Tag was introduced into umbilical cord-derived primary endothelial cell cultures (Duncan, as before), and from three additional Asian elephants and one African elephant, as well as from amniotic sac-derived epithelial cell cultures from two completely unrelated elephant donors (one Asian and one African). At 48 hours post transfection, RNA was extracted, genomic DNA was removed, and reverse transcription-PCR (RT-PCR) performed on cDNA. Primers were designed to amplify a 150–250 bp sequence from AelPyV-1 LTag (NCBI reference sequence: YP_008603286.1). These primers span the region of the virus early region gene that must be spliced out to remove a portion of the STag gene, including its stop codon. Gel bands of the expected 150–250 bp size, as well as bands migrating at approximately 400–500 bp (likely indicating expression of the STag gene), were purified and sequenced (Fig 6). The transfected cells expressed both AelPyV-1-LTag and STag, suggesting that either or both tumor antigens gene may contribute to transformation. Of potential relevance, evidence from transformation experiments using other viruses in different mammalian cell lines show that LTag alone is sufficient to transform cells [47–51]. Further work is required to determine whether either or both AelPyV-1 Tags are responsible for potential transformation of elephant cells.

### Addition of fluorescent tags may preclude T antigen expression or function

**Fig 7:**
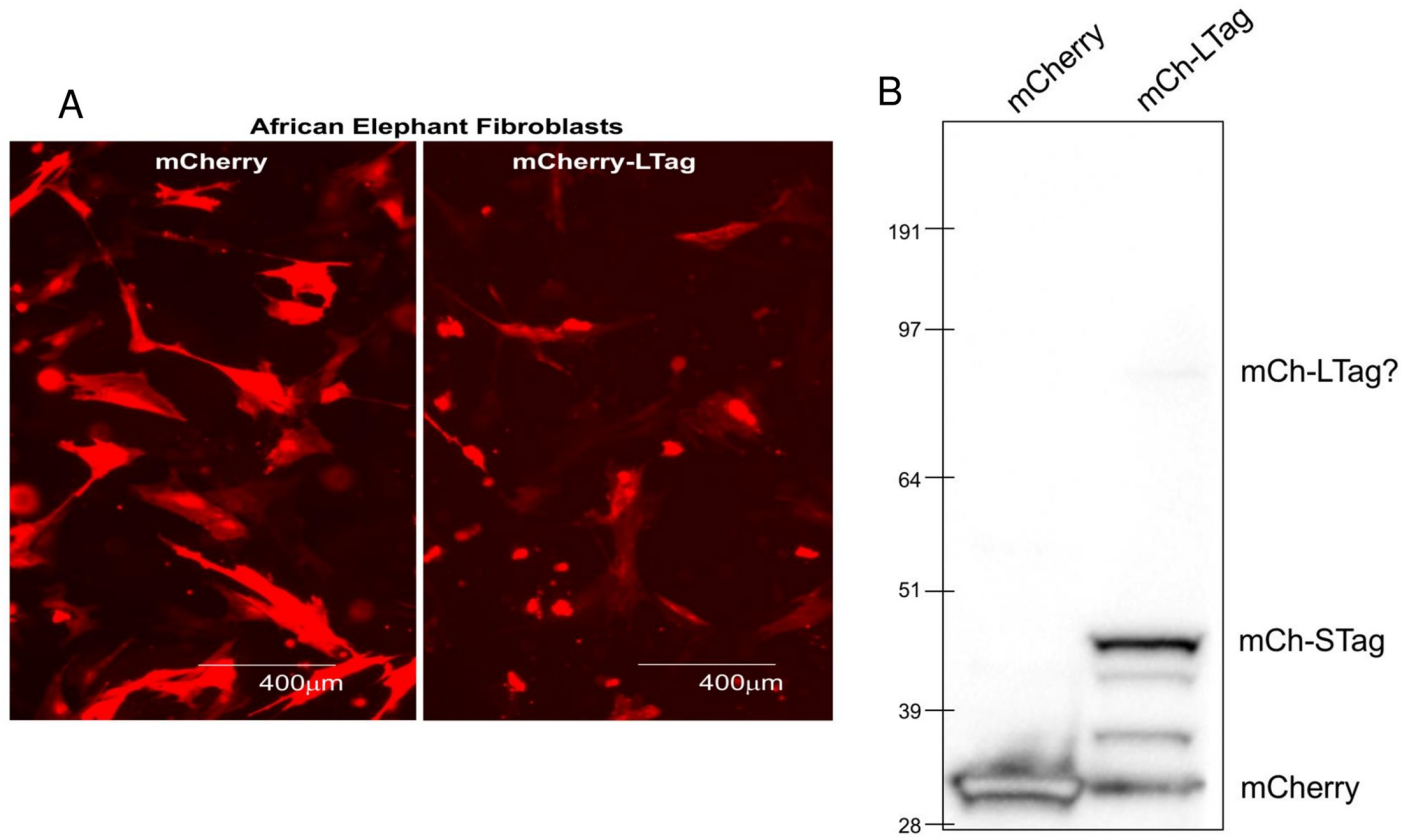
AelPyV1-LTag antigen expression in African elephant fibroblasts. **A**. African elephant fibroblasts were transfected with pAelPyV-1Tag (pcDNA3.1)+mCherry or PAelPyV-1-Tag (pcDNA3.1)+mCherry-LTag (encoding the entire early region of AeIPyV-1). 18 hours post-transfection, mCherry expression was assessed by immunofluorescence. **B**. 24 hours post-transfection, cells were harvested and lysed. Western blots were performed using antibody to mCherry. Predicted molecular weights: mCherry alone: 28.8 kDa; mCherry-STag: 48 kDa; mCherry-LTag: 104.6 kDa. The fluorescent fusion construct mCherry-LTA of original pAelPyV-1-Tag appeared to interfere with protein expression of the spliced LTag region, but not with expression of STag.

Because antibodies specific for Ae1PyV-1-LTag and STag proteins are not available, we had hoped to generate plasmids co-expressing either or both T antigens with an easily identifiable fluorescent tag (e.g., mCherry). Such labels would be particularly useful, for example, in characterizing subcellular localization of these proteins. Therefore, pAelPyV-1Tag (encoding the early region tumor antigen genes) was cloned into a plasmid to allow for the expression of amino terminally linked-mCherry. The amino terminal position of mCherry ensured that any protein (either protein encoded by the early region gene: STag or LTag) expressed after the plasmid start site would be tagged with mCherry. To determine if AelPyV-1-LTag or STag were expressed as proteins from this early region construct, primary African elephant fibroblast cells (from San Diego Zoo Global) were transiently transfected with the fluorescently tagged vector and analyzed for protein expression. Immunofluorescence revealed diffuse cytoplasmic and nuclear staining 18 h post-transfection with mCherry (Fig 7). Moreover, Western blot analysis using an antibody to mCherry showed expression of mCherry protein (28.8 kDa) in control cells transfected with the mCherry plasmid. In cells transfected with the mCherry-LTag plasmid, mCherry-tagged STag expression was observed with a band of the predicted size (48 kDa). In addition, a faint larger band was seen at the 97 kDa marker. However, this band, which likely corresponds to the mCherry-LTag, is smaller than that predicted for AelPyV-1-LTag (104.6 kDa), perhaps a result of protein degradation or interference of the mCherry tag with proper splicing of the LTag transcript.

**Fig 8:**
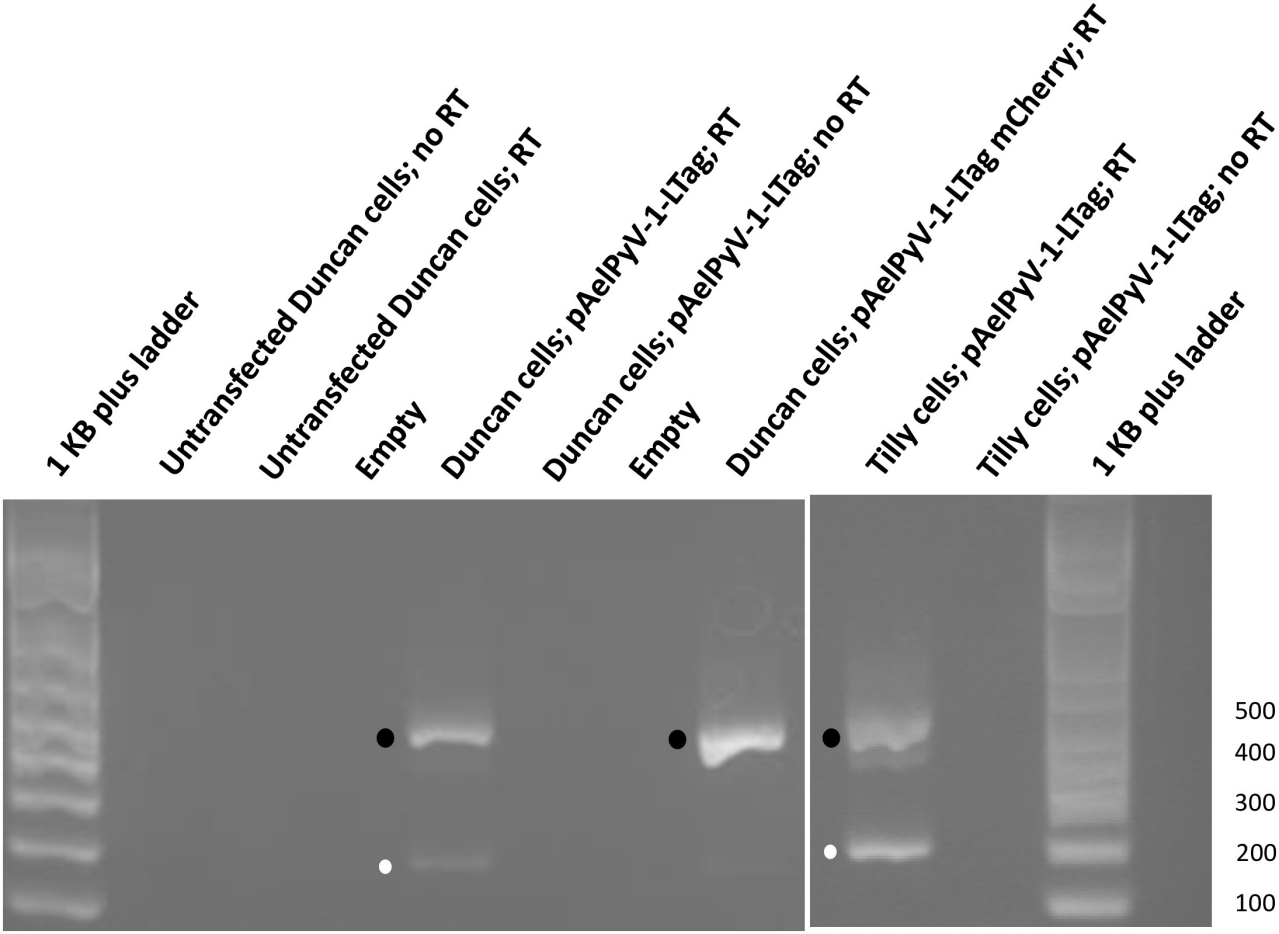
Verification of transcription of the LTag and STag antigens in transformed cells. The PAelPyV-1-Tag transfected cells expressed both LTag (expected 150–250 bp; white dot) and STag (expected 400–500 bp; black dot). Cells transfected with PAelPyV-1-Tag+mCherry-LTag expressed only STag.

To investigate if the mCherry tag interfered with proper splicing of the pAelPyV-1-Tag transcript, we transfected fresh Duncan primary endothelial cells with the fluorescent plasmid mCherry-LTag and compared them with fresh, untransfected Duncan primary endothelial cells, as well as to a transfected culture of primary endothelial cells from a second Asian elephant (Tilly). At 48 hours we performed RT-PCR on cDNA synthesized from these transfected cells. Only one 400–500 bp band was observed in the mCherry-LTag transfected cells whereas two bands (at 400–500 and 150–250 bp) were visible in the pAelPyV-1-Tag transfected positive control cells (Fig 8). Notably, cells transfected with fluorescent plasmid mCherry-LTag did not survive after 7 days under G418 selection whereas cells transfected with pAelPyV-1-Tag continued to proliferate under G418 selection.

## Discussion

In this report, we developed a plasmid, pAelPyV-1-Tag, encoding viral transforming proteins specific to elephants, and described an unique repository of primary elephant specimens. The establishment of a vector encoding elephant-specific large and small T antigens, we contend, facilitates durable transformation of primary elephant cells that will be a critical resource for not only studies of lethal herpesvirus infections in elephants but also other potential studies relevant to human cancer research. These reagents will be valuable for investigation of the intrinsic cellular response to DNA damage in elephants, as well as to characterize the functional consequences of genomic alterations unique to elephants.

Transformed elephant cell lines can be readily manipulated. For example, the contribution of individual genes can be interrogated using CRISPR/Cas9 based strategies for gene knockout or gene modification experiments. Cells with a limited lifespan may stop dividing prior to completion of the CRISPR experiments, and thus transformed lines will allow more rigorous experiments to explore the function of genes potentially involved in cancer resistance in elephants. However, it should be noted that the potential mechanism of transformation for AelPyV-1-LTag and STag have not yet been explored, nor has the immortality of cells expressing LTag and/or STag been fully confirmed. Future studies are required to determine if the LTag of this virus uses mechanisms similar to other polyomaviruses, including SV40, which expresses an LTag protein that binds and blocks the activity of two important tumor suppressor proteins (p53 and Rb), which leads to cellular transformation. If such studies reveal that the same mechanism of transformation is operative in elephant cells, then careful attention should be paid to the interpretation of results generated, particularly in studies involving the p53 and Rb pathways.

Future efforts should focus on the interaction of the synthesized large and small T antigens with cellular p53 and Rb. In the 1970s, a paradox was proposed by an epidemiologist, Richard Peto, in which he noted that increased body size (correlating with many-fold more cells, which presumably would provide more opportunities for mutations that could lead to tumors) was not consistent with the data. In other words, there was no direct relationship between cancer rates and body size. Subsequently, it was found that elephants possess 20 copies of the tumor suppressor p53, which could account for their low rates of cancer. How experimentally enforced expression of T antigens overcomes this substantial cellular safeguard to transformation may be a unique tool to evaluate tumor suppressor biology in transformed elephant cell lines.

One additional resource that this manuscript describes is a repository of archived elephant DNA samples collected from African and Asian elephants, both in the wild and in American zoos. Unlike ubiquitous models of cancer, such as mice, from which tissues can be easily collected, collection of biopsies from elephants requires extensive permitting, coordination, and skill. Even so, the authors did not have sufficient time under field conditions to collect “non-nodular” adjacent skin samples from anesthetized wild African elephants to determine if the polyomaviruses described here were specific to the nodules. This was for purely practical reasons: wild elephants can be anesthetized for only short periods of time (fewer than 45 minutes) and recover within 90 seconds of antidote administration, posing immediate danger to the veterinarians and scientists collecting biopsies. Additionally, while nodules had been observed on non-wild elephants in the past (Jacobson et al) 46, the authors were unable to find visible nodules on non-wild elephants for potential biopsy during this sample collection period.

## Supporting information

S1 Raw images (PPTX)
