# Supplementary material for: Identification of African Elephant Polyomavirus in wild elephants and the creation of a vector expressing its viral tumor antigens to transform elephant primary cells
Source: PLoS One. 2021 Feb 5;16(2):e0244334. doi: 10.1371/journal.pone.0244334 (PMC7864673; doi:10.1371/journal.pone.0244334)
Supplement: S1 Raw images — (PPTX) [file pone.0244334.s001.pptx]

## Slide 1
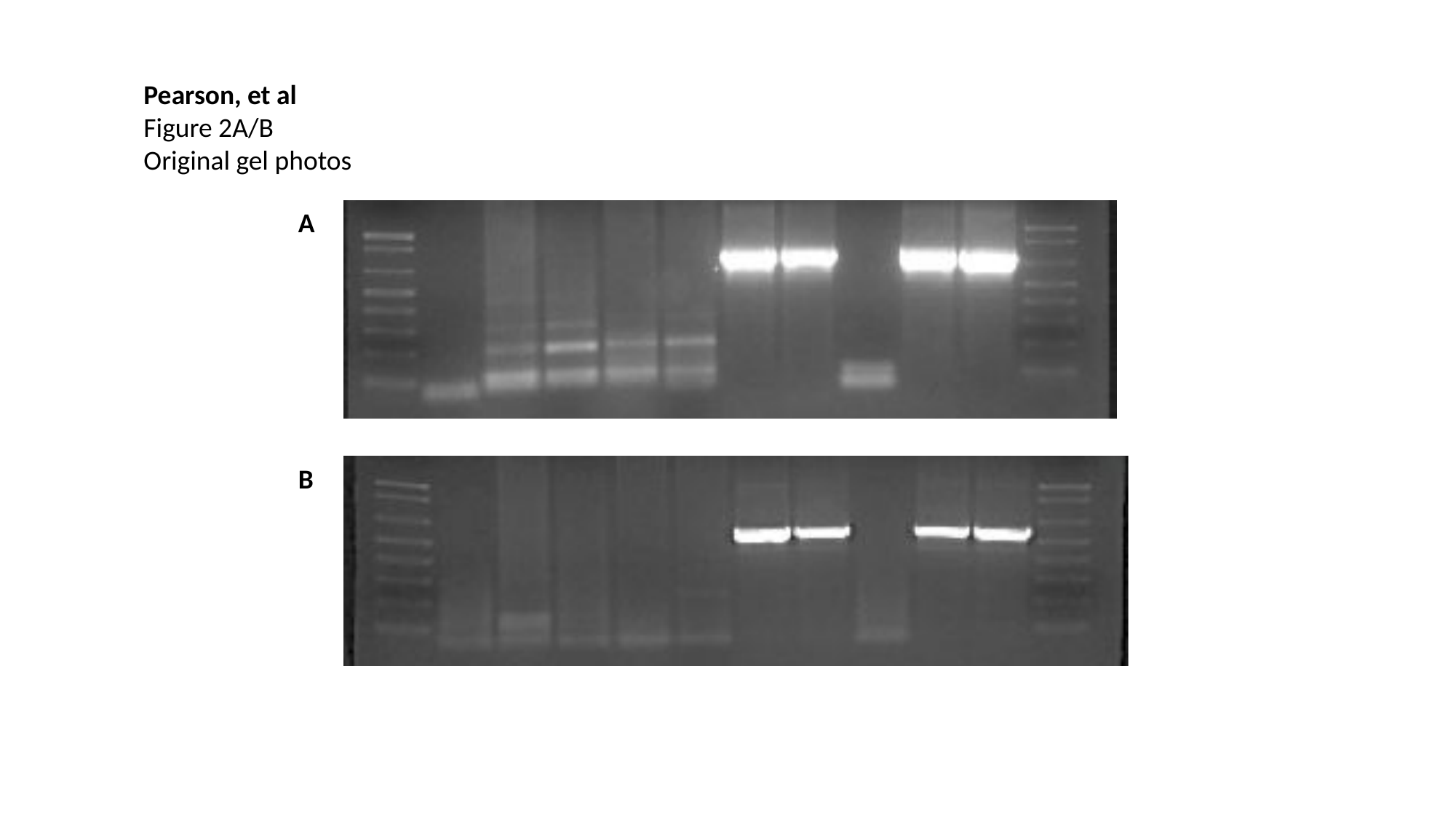

Pearson, et al
Figure 2A/B
Original gel photos
A
B

## Slide 2
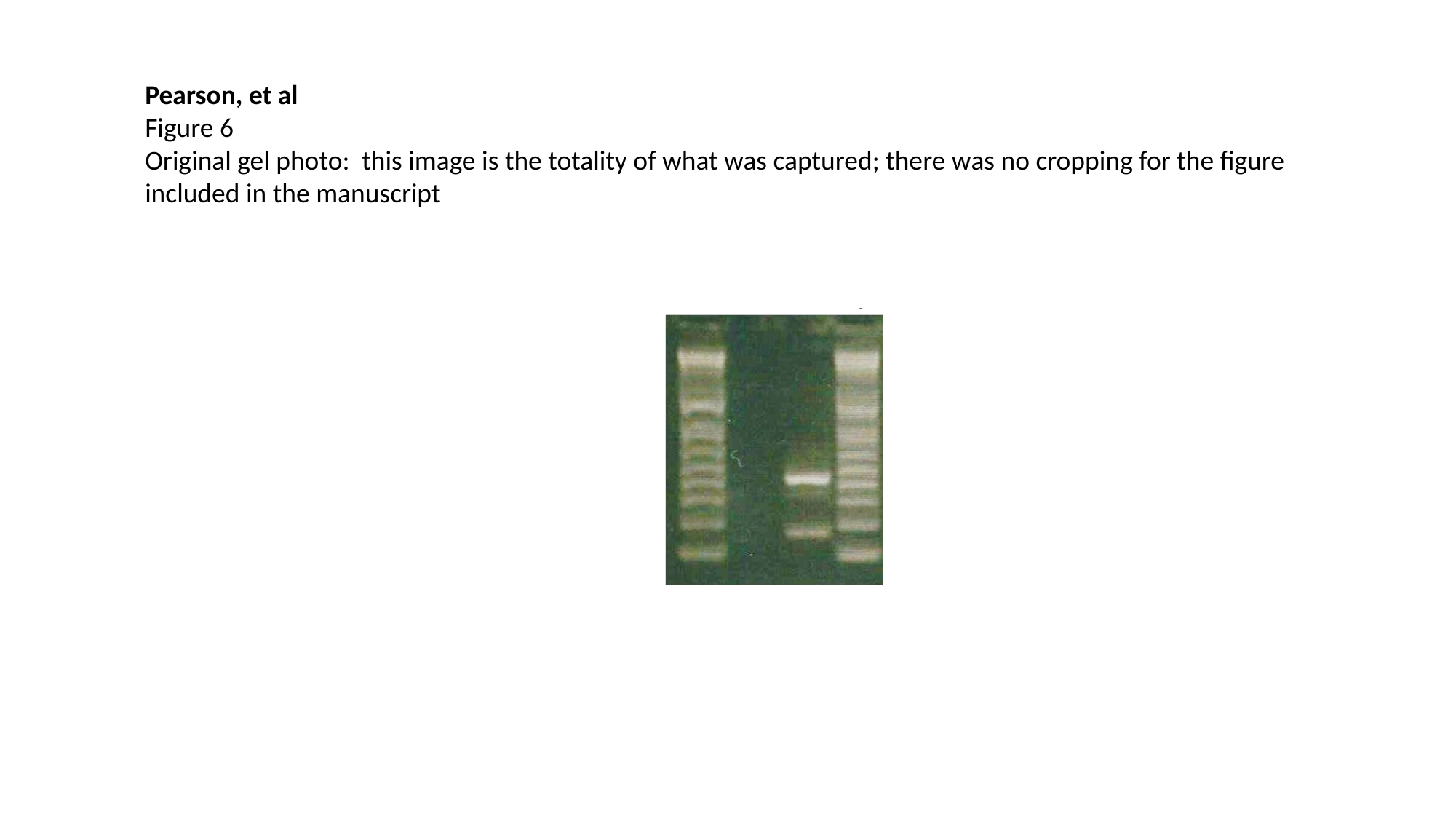

Pearson, et al
Figure 6
Original gel photo: this image is the totality of what was captured; there was no cropping for the figure
included in the manuscript

## Slide 3
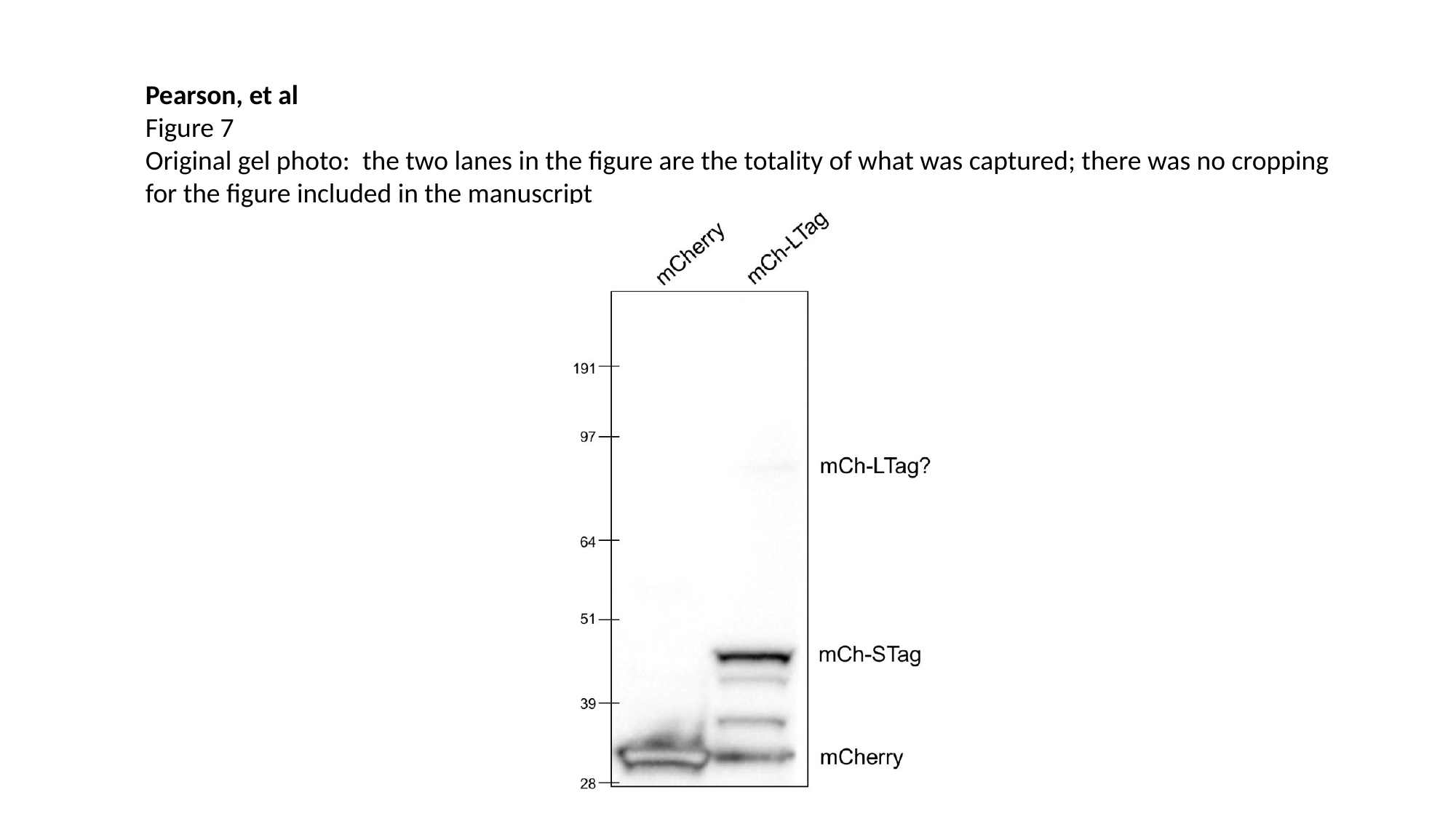

Pearson, et al
Figure 7
Original gel photo: the two lanes in the figure are the totality of what was captured; there was no cropping
for the figure included in the manuscript

## Slide 4
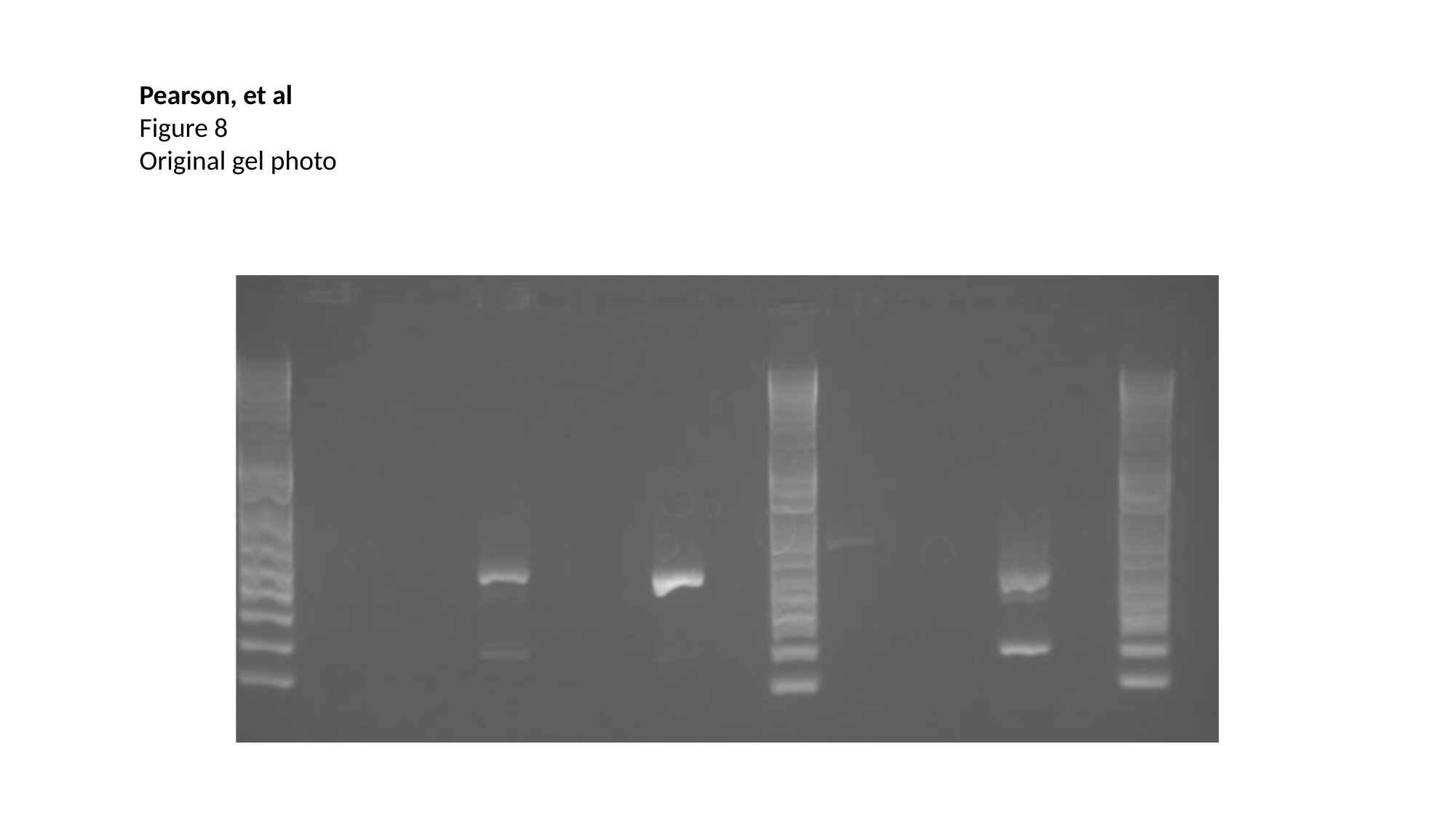

Pearson, et al
Figure 8
Original gel photo
